# Phytochemical Modulation of Ion Channels in Oncologic Symptomatology and Treatment

**DOI:** 10.3390/cancers16091786

**Published:** 2024-05-06

**Authors:** Rohan Rao, Caroline Mohammed, Lise Alschuler, Daniel A. Pomeranz Krummel, Soma Sengupta

**Affiliations:** 1 Department of Neurology & Rehabilitation Medicine, University of Cincinnati College of Medicine, Cincinnati, OH 45267, USA; 2 Department of Neurology, University of North Carolina School of Medicine, Chapel Hill, NC 27599, USA; 3 Andrew Weil Center for Integrative Medicine, University of Arizona College of Medicine, Tucson, AZ 85719, USA; 4 Department of Neurosurgery, University of North Carolina School of Medicine, Chapel Hill, NC 27599, USA; 5 Lineberger Comprehensive Cancer Center, University of North Carolina School of Medicine, Chapel Hill, NC 27599, USA

**Keywords:** phytochemicals, oncology, ion channels, voltage-gated ion channels, ligand-gated ion channels, sodium channel, potassium channel

## Abstract

**Simple Summary:**

Cancer is a leading cause of death worldwide. The costs involved in cancer diagnosis and treatment are extraordinary. Important steps that can be taken that would reduce costs include earlier cancer diagnosis for which significant headway is being made through biomarker identification in bodily fluids and imaging. Other important steps will be to identify treatment approaches that do not ‘break the bank’ and affect a patient’s wellbeing. Herein, we aim to highlight the potential of phytochemicals as a cost-effective approach to aid in the treatment of cancer. We focus on phytochemicals that target ion channels, as molecules that mediate critical communication of a cell with its environment. Ultimately, we posit that phytochemicals targeting ion channels can be employed to aid cancer treatment.

**Abstract:**

Modern chemotherapies offer a broad approach to cancer treatment but eliminate both cancer and non-cancer cells indiscriminately and, thus, are associated with a host of side effects. Advances in precision oncology have brought about new targeted therapeutics, albeit mostly limited to a subset of patients with an actionable mutation. They too come with side effects and, ultimately, ‘self-resistance’ to the treatment. There is recent interest in the modulation of ion channels, transmembrane proteins that regulate the flow of electrically charged molecules in and out of cells, as an approach to aid treatment of cancer. Phytochemicals have been shown to act on ion channels with high specificity regardless of the tumor’s genetic profile. This paper explores the use of phytochemicals in cancer symptom management and treatment.

## 1. Introduction

Plants have a fundamental role in the health and wellbeing of our lives. Importantly, they can also be utilized for their medicinal potential and are increasingly viewed as ‘medicine’, albeit of the ‘alternative’ variety. Humans have creatively employed plants as herbal medicine going back thousands of years. One of the oldest medical records, dated to 1500 B.C., is the Papyrus Ebers [1], which describes the usage of over 800 plants to treat a variety of ailments. Traditional Chinese medicine was first documented around 200 A.D. in The Divine Farmer’s Materia Medica, which describes the benefits and drawbacks of 365 different plants [1]. Through trial and error, these communities established routine protocols for the treatment of a variety of disorders by employing plants. Today, many of these same plants continue to be employed as remedies. What is loosely termed ‘Eastern medicine’ focuses primarily on a holistic approach to an individual’s illness while using natural medications. There are many potential positives to this approach. Foremost, when used appropriately, high-quality natural remedies generally produce limited side effects and are widely available at a relatively low cost [2]. Western medicine focuses more heavily on the scientific method and increasingly employs designer drugs binding with nanomolar affinity to validated, molecular targets [3]. Drugs are created with the intent of treating pathology and/or symptoms. They work by targeting specific cellular processes that contribute to an individual’s pathology. Subsequent modifications focus on increasing bioavailability and reducing off-target effects. The process of synthesizing drugs is the foundation of Western medicine.

However, the basis of modern Western medicine can trace its origins to phytochemicals. For instance, the natural opioids—morphine, codeine, and heroin—all share the same ‘parent plant’ but have undergone different manipulations. They come from the plant *Papaver somniferum*, also known as opium poppy. The plant’s usage can be dated back to the Sumerian population, who referred to it as the ‘joy plant’. People later began using opium for pain management, sedation, and disease treatment. It was centuries later that scientists realized the plant’s addictive qualities and toxicity. In the 1800s, scientists extracted morphine from the opium poppy and then further modified morphine into both heroin and codeine [4]. Both morphine and codeine are still used as anesthetics and for pain management. Another well-known use of phytochemicals in Western medicine is the derivation of penicillin from the fungus *Penicillium notatum* by Alexander Fleming in 1929. Of salience to this paper, phytochemicals further gained popular and commercial appeal in Western medicine for their initial use as chemotherapeutics. For example, the breast cancer drug paclitaxel was derived from the bark of *Taxus brevifolia* in 1962 [5]. Paclitaxel functions as a microtubule inhibitor, thereby disrupting cancer cell mitosis. The Vinca alkaloids, vincristine and vinblastine, furthered the use of phytochemicals in chemotherapy by inhibiting cancer cell proliferation in a similar mechanism to paclitaxel [6,7]. 

Although both approaches to medicine have their individual benefits, they also have their own drawbacks. Western medicine, although evidence-based, can use data from under-powered clinical trials and has a pharmaceutical bias. In addition, many therapeutics can cause side effects that are poorly tolerated [3]. The natural remedies of Eastern, or traditional, medicine can exert health benefits; however, if they are combined inappropriately, used in excess of safe dosages, adulterated with toxic compounds or used for conditions or individuals for which indications are lacking, they can lead to patient harm [3]. Due to the drawbacks in both approaches, it can be difficult to reconcile the two approaches or, for people who practice one of the approaches, to understand and agree with the other approach. Nonetheless, there is the opportunity to combine the approaches by utilizing naturally occurring compounds from nature to enhance the effects of medications, thereby improving the treatment of a variety of diseases, specifically cancer. 

Cancer is one of the leading causes of death worldwide. Although current conventional treatments are generally effective in controlling certain cancer subtypes, they can also have significant drawbacks. For instance, chemotherapy is associated with lasting debilitating side effects, such as neuropathy, hair loss, loss of appetite, fatigue, pain, and immunosuppression [8]. Another major issue with these treatments is financial toxicity [9]. There may be opportunities for the addition of natural medications to aid in the treatment of cancers. Specifically, there are compounds, or phytochemicals, found in food, herbs, and plants that exert a variety of benefits. Several phytochemicals can aid in nausea and vomiting, neuropathic pain from chemotherapy, anxiety, and dyssomnias. There is also evidence that they may help enhance the efficacy of treatments and function as cancer prophylactics [10,11,12,13,14]. Using phytochemicals to treat patients most similarly resembles practices in Eastern medicine. However, combining Western and Eastern approaches could result in more positive prognoses and make undergoing conventional treatment more bearable for patients. This review reports on the potential for a variety of phytochemicals to increase patients’ quality of life during and after treatment, as well as augment conventional treatment effects.

## 2. Ion Channels and Cancer 

Ion channels span the cell membrane of excitable cells to regulate the flux of electrically charged molecules. Ion channels are commonly classified based on what elicits their activity: ligands, voltage, or mechanical stress. Ion channels are regulated to maintain the functionality of cells, and their malfunction can result in (i.e., channelopathies) cancer. One possible effect of an unregulated ion channel is an imbalance of ions within a cell. This, in turn, may confer an electric potential that assists in the formation of a tumor and/or metastasis [15,16]. Conversely, a sustained change in electric potential has also been shown to elicit an anti-tumor response [17,18,19]. 

Ligand-gated ion channels (LGICs), or ionotropic receptors, allow for the passage of different ions following the binding of neurotransmitters. These ions include Ca^2+^, Cl^−^, K^+^, and Na^+^. LGICs are further divided into three families: purinoreceptors, Cys-loop receptors, and ionotropic glutamate receptors [16]. Purinoreceptors’ signaling molecule is adenosine 5′-trisphosphate (ATP) [20]. Due to ATP’s role in cell proliferation, when these receptors experience dysregulation they can have detrimental effects on the functionality of cells [16]. A subclass within the purinoreceptors, P2XRs are upregulated in various cancer types which can lead to a significant proliferation of cancer cells, increase cancers’ metastatic potential, and inactivate T-cells [16,21]. The Cys-loop receptors are named after the disulfide bridge contained in their extracellular domain. The Type-A GABA receptor (GABA_A_R), a prominent member of this subclass, is associated with cancer due to its possible role in cell proliferation [16]. Since these channels play a large role in both the formation and symptoms of tumors, targeting them in cancer treatment could have multifaceted benefits for the patient [16].

Voltage-gated ion channels open following a change in electric potential across the cell membrane. These channels may be selectively permeable to Ca^2+^, Cl^−^, K^+^, and Na^+^ ions. Alternatively, some voltage-gated ion channels non-selectively allow ions to pass. It has been discovered that irregular expression of the voltage-gated sodium channels (VGSCs) can be found within various tumor types [22]. In particular, the increase in intracellular Na^+^ by the α subunit of VGSCs is thought to increase solid tumor proliferation [23]. Similarly, increased expression of the voltage-gated potassium channel, Kv10.1, has been associated with faster tumor growth and increased aggressiveness compared to control tumors in animal models [24,25]. This finding was extended to human cell lines where inhibition of Kv10.1 slowed tumor growth [26]. Finally, voltage-gated Ca^2+^ channels have also been implicated in the proliferation of several tumor subtypes [27]. Most notably, the activation of membrane androgen receptors in human breast cancer cell lines led to an influx of Ca^2+^ through the Ca^2+^ channel Orai1 which was important for rapid androgen effects [28]. Given the key regulatory role that plasma membrane ion signaling has on intracellular kinase signaling, autophagy and apoptosis, these ion fluxes can promote tumors and their aggressiveness by increasing proliferation, migration, decreasing adhesion, inhibiting apoptosis, and altering various other cell signaling pathways [29,30,31,32]. Targeting these channels for the treatment of cancer could also help diminish the aggression and side effects of tumors. 

Lastly, mechanically-gated ion channels are responsible for cells’ ability to transform mechanical stimuli, like a change in pressure or touch, into electrical or chemical stimuli [33]. These ion channels also have subgroupings including the PIEZO and the OSCA/TMEM63 subtypes [33]. Although these channels have been discovered, there is little known about their pathways and how they function at a molecular level. Therefore, this review will not discuss these channels further. 

Ion channel dysregulation plays a causal role in cancer and affects an individual’s symptoms [15,16]. Certain phytochemicals can operate as inhibitors or activators of these ion channels, thereby counteracting the channels’ pro-tumorigenic effects [34]. The usage of phytochemicals could also address a variety of cancer toxicities, such as medication toxicity and financial toxicity, as natural compounds are generally well tolerated and more accessible.

## 3. Phytochemicals in Cancer Treatment

Given ion channels’ significant role in cancer pathophysiology, as outlined above, the next area of research involves identifying putative phytochemicals to selectively target ion channels to disrupt tumor growth and secondarily control symptoms. Phytochemicals are compounds derived from plants that have been shown to aid in nausea and vomiting, neuropathic pain from chemotherapy, anxiety, dyssomnias, enhance treatment effects and can function as cancer prophylactics [10,12]. Adding these compounds to treatment protocols could result in more positive prognoses and make undergoing treatment more bearable, both physically and mentally. This review will now turn its attention to new developments in research on phytochemicals directly targeting ion channels to disrupt cancer pathways. Phytochemicals mainly alter ligand-gated and voltage-gated ion channels. 

### 3.1. Anion Channel Modulators

Picrotoxin, derived from the seeds of the fishberry shrub *Anamirta cocculus*, is a potent antagonist of GABA_A_Rs (Figure 1). As such, it prevents the flux of chloride anions through GABA_A_Rs and thus acts as a stimulant with the potential to induce tonic-clonic seizures (Table 1) [35]. It was listed in the *Merck Index* as early as the 1970s as a barbiturate overdose antidote [36,37]. Regarding anti-tumor activity, it is thought to act as an immunomodulator. Current trends in cancer immunotherapy focus on the inherent tumor-suppressive effects of cancers by providing PDL-1 inhibition or CTLA4 antagonism. Picrotoxin has been shown to inhibit a mouse model of colorectal cancer growth by enhancing the cytotoxic effects of tumor-infiltrating CD8+ T-cells via the antagonism of GABA_A_Rs [38,39,40]. The hypothesis is that the secretion of GABA promotes an immune-tolerant state permissive of tumor growth. Picrotoxin has also been studied as an anti-tumor agent in human prostate cancer cell lines [41,42,43]. Wu et al. identified that GABAergic signaling mediated EGFR-Src pathway activation and that administration of picrotoxin inhibited prostate cancer growth through inhibition of this pathway [42]. Picrotoxin has also been studied in pancreatic cancer. To understand its mechanism in this tumor subtype, it is important to note that GABA can have an excitatory mechanism by promoting an influx of calcium or efflux of chloride. This ‘GABA switch’ has been well studied in the developing brain. Takehara et al. revealed that in pancreatic cancer cells, the activation of GABA_A_Rs leads to calcium influx and activation of pro-tumorigenic intracellular pathways such as the MAPK/ERK pathway [44,45]. Administration of picrotoxin inhibited this GABA-mediated MAPK/ERK activation. Moreover, in human melanocyte/keratinocyte cocultures, administration of picrotoxin (100 µmol/L) inhibited intercellular GABA signaling and decreased keratinocyte “switching” to a pro-tumorigenic phenotype [46].

Similar to picrotoxin, bicuculline is an alkaloid GABA antagonist derived from the plant *Dicentra cucullaria* [44,49]. Its mechanism of action was first elucidated in the 1970s in the spinal cord of cats under anesthesia [50,51]. Bicuculline, like picrotoxin, can be used clinically as a barbiturate reversal agent with the potential to induce tonic-clonic seizures in vivo [51]. In vitro, bicuculine inhibited the proliferation of human pancreatic cancer cells through antagonism of the EGFR-Src pathway detailed above [44]. Unlike picrotoxin, bicuculline has not been extensively studied in relation to tumor immunosuppression which could serve as a future direction for study. The multifactorial mechanism of action could potentially make bicuculline, picrotoxin, and other GABA_A_R antagonists potent anti-cancer agents. Conversely, this lack of specificity makes picrotoxin a potent epileptogenic at high doses [52]. Future directions to allow for GABA_A_R antagonists to be safely used in cancer will be to prevent off-target effects as well as penetration to the CNS where they are so potent. Alternatively, modulating the function of the receptor may offer a successful approach. For example, the synthetic GABA_A_R-positive allosteric modulator, QHii066, is highly specific and has been shown to impair tumor cell viability alone as well as sensitize medulloblastoma subtypes, melanoma, and lung adenocarcinoma to radiation [53,54,55,56,57,58]. 

### 3.2. Cation Channel Modulators

**Figure 2:**
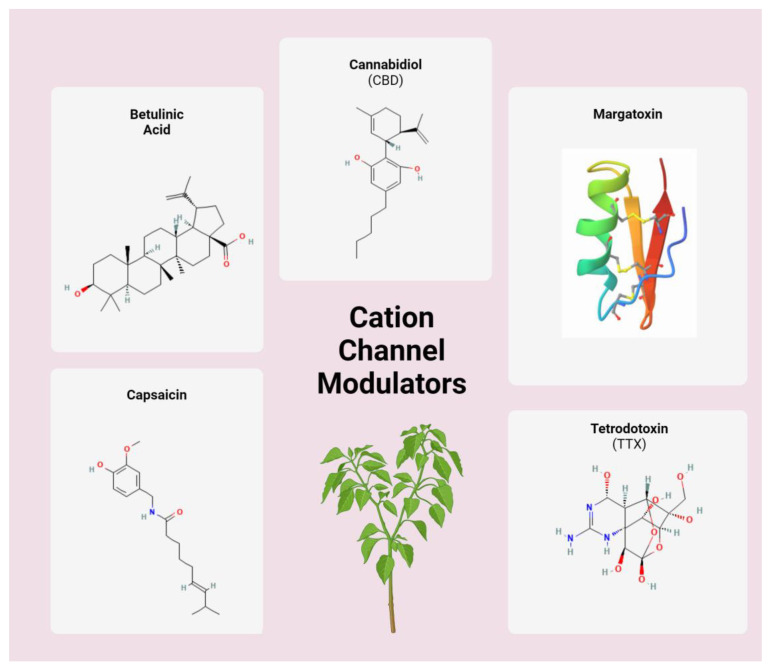
Structures of select cation channel-modulating phytochemicals. Molecular structures provided by NIH PubChem and RCSB PDB [59,60,61,62,63,64,65]. Shown is the pepper plant from which capsaicin is derived.

Betulinic acid is a pentacyclic triterpene derived from a variety of plants such as birch, eucalyptus, and plane trees (Figure 2) [66]. It is most readily isolated from white birch (*Betula pubescens*) where its extraction rate is maximized at 2–3% [66,67]. White birch, and thus betulinic acid, has been documented in Native American folk medicine [68]. Betulinic acid has been shown to antagonize N- and T-type calcium channels [69,70]. It has been shown to be effective against many types of cancers in vitro, including GI, pancreatic, myeloid leukemia, and melanoma [71,72]. It is thought to induce a pro-apoptotic state in cancer cells through mitochondrial depolarization [73]. However, further research is needed on whether the antagonism of calcium channels is the mechanism by which betulinic acid induces the mitochondrial (intrinsic) apoptosis pathway [72]. 

**Figure 3:**
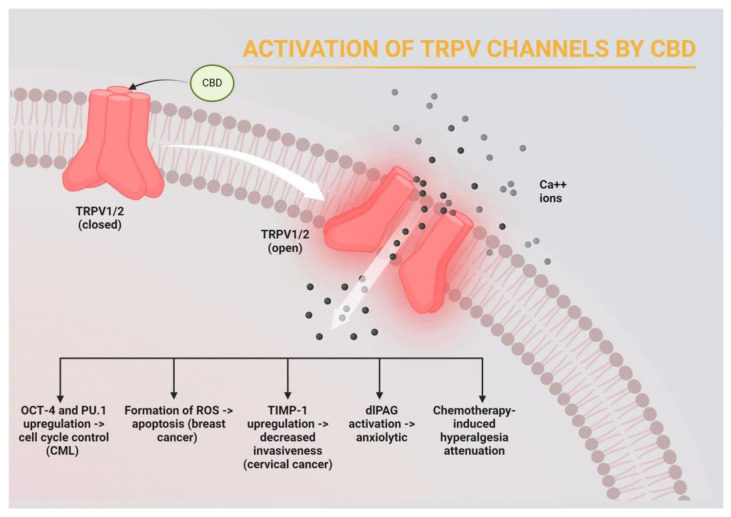
Example of how modulation of an ion channel can impact cancer treatment and symptom management. Cannabidiol (CBD) binds to the TRPV receptor, eliciting an influx of calcium cations. This in turn leads to intracellular downstream effects, both in cancer cells and somatosensory cells. The mechanism by which TRPV activation inhibits cancer pathogenesis appears to vary by tumor subtype. OCT-4, Octamer-binding transcription factor 4; PU.1, Purine-rich binding transcription factor; ROS, reactive oxygen species; TIMP-1, Tissue inhibitor matrix metalloproteinase 1; dIPAG, dorsolateral periaqueductal gray.

Cannabidiol (CBD) is the main constituent of the plant *Cannabis sativa* [74]. CBD is favored in clinical practice as it avoids the potentially toxigenic and psychogenic effects of tetrahydrocannabinol (THC). Its analgesic, anxiolytic, and antiemetic effects are well documented. It is anecdotally used as an appetite stimulant especially for cancer patients undergoing systemic chemotherapy though the data to support this usage are limited [75]. Regarding its antitumor effects, CBD has been shown to alter multiple intracellular pathways. For example, in a mouse model of breast cancer, CBD induces endoplasmic reticulum stress to induce apoptosis, though this is not an ion channel-mediated anti-cancer approach [76]. CBD binds to the CB1 and CB2 receptors which are G-protein-coupled receptors found on neurons [77]. However, CBD also has off-target effects on transient receptor potential vallinoid (TRPV) receptors which function as non-selective cation channels in the plasma membrane to regulate calcium release intracellularly (Figure 3) [74,78]. Resulting from its effect on ion channels, CBD-mediated dysregulation of cytoplasmic calcium can lead to increased production of reactive oxygen species, apoptosis, and ER stress [79]. In breast cancer cell lines, TRPV activation by CBD led to increased intracellular calcium concentrations and the formation of reactive oxygen species, inducing apoptosis [80]. In chronic myeloid leukemia, activation of TRPV via CBD inhibited proliferation and cell cycle progression in vitro [81]. CBD treatment of human cervical cancer cells reduced cancer invasion as quantified by the Matrigel invasion assays [82]. The proposed mechanism in this study is that TRPV activation by CBD led to upregulation of tissue inhibitor of matrix metalloproteinases-1 (TIMP-1) and subsequent decreased invasiveness [82]. This finding was reversed by antagonism of TRPV and siRNA silencing of TIMP-1 which provides further support for this mechanism. CBD will also be described further below in its more well known role in modulating cancer symptomatology. 

Margatoxin (MgTX) is a 39 residue (4.2 kDa) peptide isolated from *Centruroides margaritatus* [83]. Although not a traditional phytochemical as it is not plant-derived, MgTX is derived from a ‘natural’ source and acts on ion channels, making it pertinent to this review. MgTX inhibits ion channel subtype Kv1.3 [84]. This channel subtype has been thought to be overexpressed in several cancer subtypes [85]. It is thought that Kv1.3 induces cancer pathogenesis by regulating cell cycle progression through upregulating the expression of cyclin or down-regulating cyclin-dependent kinase inhibitors [86]. Jang et al. xenografted A549 cells (a human lung adenocarcinoma cell line) onto nude mice. Subsequent Kv1.3 inhibition by MgTX led to a significant decrease in tumor volume by increasing p21 levels and decreasing Cdk4 and cyclin D3 [87,88]. This suggests the antitumorigenic mechanism of MgTX is through cell cycle inhibition. Jang et al. furthered the prior work by Fraser et al. which studied the effects of MgTX on an in vitro rat prostatic cancer cell line [89]. The conclusion was that MgTX reduced the prostate cancer cell line proliferation in a dose-dependent manner. The novelty of Jang et al.’s study was that it was an in vivo study using a xenograft model. 

Tetrodotoxin (TTX) is a guanidium compound produced by bacteria in puffer fish and other marine animals [90,91]. Like MgTx, we include TTX given its derivation from a ‘natural’, albeit not plant, source. TTX antagonizes voltage-gated sodium channels (Na_V_) isoforms [92]. Na_V_ is thought to play a role in cancer metastasis via the upregulation of the EGFR signaling pathway particularly in human non-small cell lung cancer [59]. Furthermore, the upregulation of sodium channel subtype Na_V_1.7 has been shown to be associated with the metastatic potential of prostate cancer in vitro and in vivo [93]. Similarly, subtypes Na_V_1.1 and Na_V_1.6 have been implicated in colorectal cancer lymph node metastasis [94]. In cervical cancer, Na_V_1.6 has been shown to be upregulated. Given the above data showing upregulation of voltage-gated sodium channel subtypes modulating cancer metastasis, it has been a logical next step to look for inhibitors of this process. Hernandez-Plata et al. blocked VGSC of cervical cancer cells in vitro with TTX and confirmed channel blockade via electrophysiology [95]. While TTX treatment did not impact proliferation, it did significantly reduce invasiveness. In a non-small cell lung cancer cell line, TTX administration reduced cell invasion by up to 50% [96]. Few in vivo studies have analyzed TTX. In Ehrlich ascites carcinoma-bearing mice, treatment with TTX led to a significant decrease in tumor weight and a significant increase in survival compared to control [97]. Furthermore, this study used doxorubicin as a positive control group which also emphasized the improved side effect profile of TTX. 

Capsaicin is the primary ingredient in chili peppers and functions as a TRPV1 agonist [98]. As mentioned previously, TRPV1 is a non-selective cation channel mediating the intracellular flow of calcium ions [98]. In SUM149PT cells, a model of triple-negative breast cancer, stimulation of TRPV1 by capsaicin led to a significant increase in intracellular calcium [99]. Subsequently, activation of TRPV1 by capsaicin caused inhibition of cancer cell growth by inducing apoptosis and necrosis. Similarly, in 5637 cells modeling urothelial cancer, TRPV1-expressing cells treated with capsaicin led to a capsaicin-mediated intracellular calcium increase with subsequent growth inhibition, apoptosis, and migration inhibition [100]. Prostate carcinoma (PC-3, Du 145, LNCaP) cell lines transfected with TRPV1 cDNA and subsequently treated with low-dose capsaicin caused mitochondrial calcium accumulation and apoptosis [101]. Finally, activation of TRPV1 channels in papillary thyroid cancer BCPAP cells by capsaicin inhibited the metastasis of this cell line through the downregulation of epithelial–mesenchymal transition transcription factors [102]. However, this paper did not completely connect the increase in intracellular ion flow with the downregulation of the transcription factors. Capsaicin also appears to have a multitude of non-TRPV1 targets providing it multiple, independent pathways to inhibit tumor growth [103,104,105].

**Table 1:** Select phytochemicals that modulate ion channels for tumor treatment and symptom management.

Chemical	Mechanism	Cancer Subtype(s)	Citations
Picrotoxin	GABA antagonist; Immunomodulator; EGFR-Src pathway inhibition; MAPK/ERK inhibitor	Prostate, colorectal, pancreatic cancers, and melanoma	[42,43,44,49]
Bicuculline	GABA antagonist; EGFR-Src pathway inhibition	Pancreatic cancer	[44,51]
Betulinic acid	Inhibition of N- and T-type Ca^2+^ channels; modulation of intracellular mitochondrial apoptotic pathways	Gastrointestinal and pancreatic cancers, myeloid leukemia	[71,72]
Cannabidiol	TRPV agonist; intracellular calcium disruption	Chronic myelogenous leukemia, breast, cervical, and lung cancers	[79,80,81,82,106]
Margatoxin	Kv1.3 potassium channel inhibitor; cell cycle regulation	Lung adenocarcinoma	[87,89]
Tetrodotoxin	Na_V_ inhibitor; modulating cancer metastatic potential	Non-small cell lung, colorectal, and prostate cancers	[95,96,97]
Capsaicin	TRPV1 agonist; intracellular calcium disruption	Triple-negative breast, urothelial, prostate, papillary thyroid cancers	[99,100,101,107]

## 4. Phytochemicals Modulating Cancer Symptomatology 

One of the most common and aggravating symptoms of cancer is cancer-related pain (CRP). Up to 70% of cancer patients have CRP either directly related to tumor burden or associated with treatment side effects [108]. Regarding the second mechanism, many chemotherapeutics have a side effect of neuropathy which can result in neuropathic pain if allowed to become chronic (Table 2). 

One of the suspected mechanisms driving nociception is that the release of ATP from apoptotic cells leads to the activation of P2X receptors on adjacent cells. Given that P2X receptors are excitatory and calcium-permeable, they help maintain nociceptive signals [109]. Resveratrol is a natural polyphenol found in peanuts, mulberries, grapes, and red wine [110,111]. Resveratrol has also been shown to increase the thermal and mechanical hypersensitivity threshold in a rat HIV model of chronic neuropathic pain through the downregulation of P2X receptors [109,110]. Another potential mechanism of action of resveratrol is through sodium ion modulation. Jia et al. showed that resveratrol decreased Nav1.7 expression and subsequently decreased neuropathic pain in a rat model of chronic constriction injury [112]. In human cells, resveratrol has been shown to suppress pancreatic cancer cell proliferation by inactivation of AKT-GSK3β and ERK1/2 signaling [6,113]. Similarly, there is preliminary evidence that resveratrol induces calcium influx in human mesothelioma cell lines, providing another possible mechanism of resveratrol’s anti-cancer properties [114]. However, the poor bioavailability of resveratrol limits its use [6,115]. Regardless, resveratrol serves as a potent phytochemical in cancer treatment as it theoretically inhibits cancer proliferation and serves to manage nociception via LGIC regulation.

Similarly, puerarin, isolated from *Radix puerariae*, decreases the expression of P2X3 receptors in the dorsal root ganglion in rats [116]. This in turn leads to decreased thermal and mechanical hypersensitivity. While this study did not directly study a cancer animal model, it provided useful preliminary information on the analgesic effects of puerarin. Another possible mechanism for puerarin regulating pain is through the inhibition of voltage-gated sodium channels in the dorsal root ganglia (DRG). Zhang et al. treated Sprague-Dawley rats with paclitaxel to create an animal model of chemotherapy-induced neuropathic pain [117]. Subsequent application of puerarin reduced excitability and blocked VGSC in rat DRGs [117]. This in turn led to decreased pain perception in rats as quantified by a series of behavioral tests. Interestingly, puerarin had a stronger blocking effect on the TTX-resistant Nav1.8 channel than TTX, which suggests that puerarin may have a use in pain resistant to TTX. Puerarin also modulates other chemotherapeutic side effects through non-ion channel mechanisms [118]. For example, 5-fluorauracil (5-FU) associated intestinal mucositis has been shown to be improved through JAK inhibition after puerarin administration [119].

CBD has become a popular adjuvant treatment in cancer patients for symptoms such as nausea, vomiting, anxiety, decreased appetite, and pain management [120]. CBD modulates some of these symptoms through CB1 and CB2 receptors which are GPCRs and thus outside the scope of this paper. As such, we will focus on the anxiolytic and analgesic effects of CBD as these are thought to be mediated by TRPV ion channels as described above [80,81,121]. Campos et al. treated Wistar rats with CBD directed at the dorsolateral periaqueductal gray (dlPAG) via cannula [122]. The dlPAG is a midbrain structure thought to be involved in the control of anxiety. Administration of high doses of CBD to the dlPAG and subsequent TRPV1 activation led to increased maze exploration which is a rat behavioral model used to assess anxiety. CBD is also well known for its analgesic effects which has enabled it to be an adjunctive treatment for cancer-associated pain [123,124]. This anti-hyperalgesia effect is thought to be modulated by activation of the TRPV1 receptor, as the hyperalgesia effect is reversed when CBD is administered with the selective TRPV1 antagonist capsazepine [121,123,124].

In addition to its anti-cancer effects, TTX has a potent role in ameliorating cancer-associated pain [91]. As mentioned previously, TTX antagonizes VGSC. Prior research has shown that VGSC subtype Na_V_1.3 is massively upregulated in peripheral nerves following nerve injury in a patient model of neuropathic pain [125,126]. The VGSC subtype Na_V_1.7 has been shown to be significantly upregulated in human DRG neurons in cultures treated with paclitaxel to mimic neuropathic pain [127]. Given reported VGSC upregulation in pain models, it seems intuitive to turn to TTX as a treatment. However, the research in cancer chemotherapy-induced neuropathic pain is somewhat contradictory. Intraperitoneal administration of TTX did not attenuate pain induced by vincristine treatment [128]. In contrast, Alvarez and Levine found that intramuscular administration of TTX significantly reduced neuropathic pain in a rat model of oxaliplatin-induced neuropathic pain [129]. Nieto et al. similarly found that subcutaneous TTX at doses of 3–6 µg/kg attenuated cold and mechanical allodynia in a mouse model of neuropathic pain induced by paclitaxel without toxicity or motor incoordination [130]. The variation in TTX-mediated attenuation of allodynia in the three previous studies could possibly be attributed to varied mechanisms by which the three chemotherapies induce neuropathic pain.

Like picrotoxin and bicuculline, TTX is known for its potentially toxic effects on humans, even at low doses. TTX’s safety profile has been preliminarily supported in humans by Hagan et al. on patients with cancer-related pain refractory to opiates and other analgesics [131,132,133]. In total, 30 µg TTX was given subcutaneously twice daily for four days with ~50% of patients having a significant decrease in pain intensity and no evidence of severe cumulative toxicity or tolerance [131]. Similarly, Goldlust et al. gave patients with chemotherapy-induced neuropathic pain subcutaneous TTX in dosages ranging from 15 to 60 µg daily for 4 consecutive days [134]. Cumulative responder analysis showed a significant decrease from placebo in the 30 µg BID group, improvement in secondary quality of life metrics, and minimal toxicity. This suggests that the therapeutic dose of TTX is less than the toxic dose in humans with short-term administration [135].

**Table 2:** Examples of phytochemicals that modulate ion channels for symptom management.

Chemical	Mechanism	Symptom(s)	Citation(s)
Resveratrol	P2X receptor inhibitor, sodium channel agonist	Pain management	[42,111,112,114]
Puerarin	Decreased P2X receptor expression; Na_V_ inhibitor; EGFR-Src pathway inhibition	Chemotherapy-induced neuropathic pain	[116,117,118]
Cannabidiol	TRPV activation; intracellular calcium disruption	Pain management, anxiolytic	[79,80,121,122,123,124]
Tetrodotoxin	Na_V_ inhibitor	Pain management	[91,129,130,131,132,133,134,135]

## 5. Conclusions and Future Directions

In this review we have highlighted phytochemicals that modulate ion channel function and alter intracellular concentrations of anions and cations, thereby modifying cancer invasiveness, proliferation, and migration. We have detailed several mechanisms by which phytochemical-mediated modulation of ion channels may not only directly impact cancer treatment but also symptom management. Modern chemotherapies offer a broad approach to cancer treatment but, unfortunately, are associated with a host of side effects including neuropathy, pancytopenia, nausea, and fatigue. Positively, the advent of precision oncology, or the molecular profiling of tumors for target identification, has reduced the occurrence of off-target effects and some associated side effects [136]. While targeting of ion channels to modulate intracellular ion concentrations is also anticipated to progress. Certainly, phytochemicals can serve as a natural approach by targeting these ion channels. A significant challenge, however, is demonstrating the efficacy of phytochemicals, which would be needed to perform phase I clinical trials to determine the safety profiling. There is great potential value in these trials as several phytochemicals have simultaneous anti-tumorigenic and symptom control mechanisms. A logical approach moving forward would be to integrate a phytochemical(s) as a therapeutic ‘add-on’. Careful thought must be applied to the design of such trials to maximize their potential use. A few of the phytochemicals discussed above may have prophylactic potential to prevent tumor metastasis. For example, tetrodotoxin (TTX) blockage of VGSC led to decreased invasiveness of non-small cell lung cancer in vitro [96]. Therefore, treatment with TTX may need to occur prophylactically rather than therapeutically for it to have its most significant effect. 

An additional concern with phytochemicals is the unknown side effect profile given limited clinical trial data. For example, the CBD-induced generation of ROS via TRPV1 activation could affect both non-tumor and tumor cells equally at anti-neoplastic doses. Indeed, this has been observed with potential antineoplastic plant extracts showing promise at doses that generate antineoplastic effects in man but may have intolerable toxicity due to their non-selectivity (i.e., laetrile, graviola, artemisinin) [137,138,139]. Another example, picrotoxin has shown anti-cancer effects; however, there is a high potential for epileptic side effects at possible therapeutically impactful dosages. Research in animal models showed an average dose of 3–10 mg/kg of picrotoxin delivered intraperitoneally was sufficient to induce seizures [52]. In such cases, the risks may outweigh the clinical benefits. However, clinical trials involving TTX have shown that therapeutic doses are associated with minimal side effects such as oral paresthesia and oral hypoesthesia [131,132,133,134]. Given the many reports of the potential benefits of phytochemicals to cancer treatment, progress may be made in moving them to the clinic not ‘as is’ but by searching for similar chemical compounds amongst our vast knowledge base that may be more specific. For example, employing an in silico approach to use the backbone of phytochemicals to search established drug libraries to screen for anti-cancer candidates. Similarly, the identification of new active compounds may be expedited by employing NMR spectroscopy on unfractionated phytochemical compounds followed by comparison to molecular networking platforms [117,118]. 

Much of Western medicine is based on plant-derived chemicals and the earliest chemotherapies are also plant-derived [4,5,6,7]. However, there has been a significant slowing in phytochemical drug discovery for several reasons, including difficulty extracting high volumes of active ingredients, structural rigidity of phytochemicals, and difficulty gaining intellectual property over naturally-occurring chemicals [140]. Returning to phytochemicals for more specific modulation of ion channels is a promising field in oncology. 

## Figures and Tables

**Figure 1:**
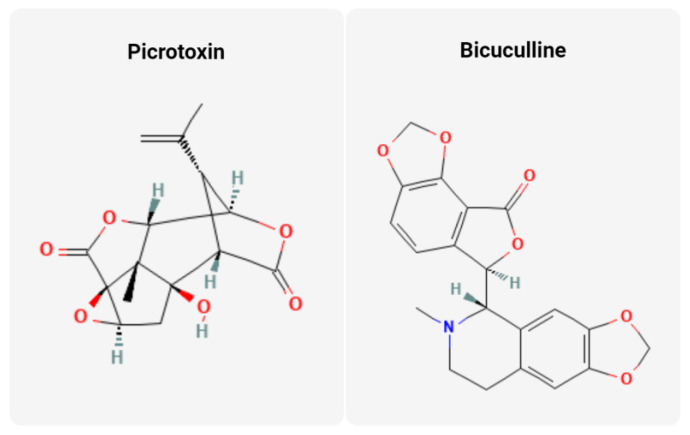
Structures of two classic anion channel-modulating phytochemicals. Molecular structures of picrotoxin and bicuculline provided by NIH PubChem [47,48].
